# Blood Markers in Healthy-Aged Nonagenarians: A Combination of High Telomere Length and Low Amyloidβ Are Strongly Associated With Healthy Aging in the Oldest Old

**DOI:** 10.3389/fnagi.2018.00380

**Published:** 2018-11-28

**Authors:** Gorka Fernández-Eulate, Ainhoa Alberro, Maider Muñoz-Culla, Miren Zulaica, Mónica Zufiría, Myriam Barandiarán, Igone Etxeberria, José Javier Yanguas, Maria Mercedes Gallardo, Nora Soberón, Ana María Lacosta, Virginia Pérez-Grijalba, Jesús Canudas, Noelia Fandos, Pedro Pesini, Manuel Sarasa, Begoña Indakoetxea, Fermin Moreno, Itziar Vergara, David Otaegui, Maria Blasco, Adolfo López de Munain

**Affiliations:** ^1^Department of Neurology, Donostia Universitary Hospital, San Sebastián, Spain; ^2^Neurosciences Area, Biodonostia Health Research Institute, San Sebastián, Spain; ^3^Department of Personality, Assessment, and Psychological Treatments, Faculty of Psychology, University of the Basque UPV/EHU, San Sebastián, Spain; ^4^Matia Fundazioa, San Sebastián, Spain; ^5^Telomeres & Telomerase Group, Molecular Oncology Programme, Spanish National Cancer Research Center, Madrid, Spain; ^6^Araclon Biotech, Zaragoza, Spain; ^7^Primary Health Area, Biodonostia Institute, San Sebastián, Spain; ^8^Health Services Research on Chronic Patients Network, REDISSEC, Bilbao, Spain; ^9^Telomeres & Telomerase Group, Molecular Oncology Programme, Spanish National Cancer Research Center, Madrid, Spain; ^10^Centro de Investigación Biomédica en Red de Enfermedades Neurodegenerativas (CIBERNED), Instituto Carlos III, Madrid, Spain; ^11^Department of Neurosciences, University of the Basque Country, San Sebastián, Spain

**Keywords:** aging, composite neurologically healthy aging score, telomere, Amyloid-β, elders

## Abstract

Many factors may converge in healthy aging in the oldest old, but their association and predictive power on healthy or functionally impaired aging has yet to be demonstrated. By detecting healthy aging and in turn, poor aging, we could take action to prevent chronic diseases associated with age. We conducted a pilot study comparing results of a set of markers (peripheral blood mononuclear cell or PBMC telomere length, circulating Aβ peptides, anti-Aβ antibodies, and ApoE status) previously associated with poor aging or cognitive deterioration, and their combinations, in a cohort of “neurologically healthy” (both motor and cognitive) nonagenarians (*n* = 20) and functionally impaired, institutionalized nonagenarians (*n* = 38) recruited between 2014 and 2015. We recruited 58 nonagenarians (41 women, 70.7%; mean age: 92.37 years in the neurologically healthy group vs. 94.13 years in the functionally impaired group). Healthy nonagenarians had significantly higher mean PBMC telomere lengths (mean = 7, *p* = 0.001), this being inversely correlated with functional impairment, and lower circulating Aβ40 (total in plasma fraction or TP and free in plasma fraction or FP), Aβ42 (TP and FP) and Aβ17 (FP) levels (FP40 131.35, *p* = 0.004; TP40 299.10, *p* = 0.007; FP42 6.29, *p* = 0.009; TP42 22.53, *p* = 0.019; FP17 1.32 *p* = 0.001; TP17 4.47, *p* = 0.3), after adjusting by age. Although healthy nonagenarians had higher anti-Aβ40 antibody levels (net adsorbed signal or NAS ± SD: 0.211 ± 0.107), the number of participants that pass the threshold (NAS > 3) to be considered as positive did not show such a strong association. There was no association with ApoE status. Additionally, we propose a “*Composite Neurologically Healthy Aging Score”* combining TP40 and mean PBMC telomere length, the strongest correlation of measured biomarkers with neurologically healthy status in nonagenarians (AUC = 0.904).

## Introduction

Aging affects all body tissues, increasing susceptibility to disease and inexorably leading to a decline in health. Many factors, from the activation or inactivation of genes to the alteration of biochemical pathways due to environmental changes, appear to be involved in aging (López-Otín et al., 2013; Maynard et al., 2015); although the relative impact of each factor may be different at each range of age. While familial and genetic contributions to aging may be modest in survival rates up to the mid-eighties (Fraser and Shavlik, 2001), from the ninth to the eleventh decade, longevity seems to be strongly influenced by genetic factors (Perls et al., 2002; Atzmon et al., 2005). Research in centenarians, however, has provided evidence that a genetic contribution to human longevity is decisive but not exclusive. So far, relatively few specific genetic variants related to longevity have been identified (Christensen et al., 2006; Wheeler and Kim, 2011), and even when compared with younger individuals, centenarians share similar disease-associated variants, underlying the importance of other unknown factors like the environment, that may be operating through epigenetic pathways (Sebastiani et al., 2012). People older than 80 years of age, the so-called “Oldest old,” are the fastest growing segment of the population in developed regions. Despite improvements in life expectancy, The 2015 World Report On Aging And Health of the WHO outlines the importance of fostering healthy aging in elders (World Health Organization, 2015). This emphasizes the need to study risk factors that may converge in poor aging, neurodegeneration and functional impairment. From a clinical perspective, poor or frail aging is strongly associated with neurological impairment (both motor and cognitive) and incident dementia (Kulmala et al., 2014), and predisposes individuals to falls, hospitalization, disability, loss of autonomy (dependence) and death.

Most neurodegenerative conditions are age-related (Niccoli and Partridge, 2012), and although aging itself is not a disease, the age-dependent structural changes and loss of functions may lead to different chronic diseases, been Alzheimer's disease (AD) one of the most prevalent. Aged brains show changes, like protein deposition, even in the absence of clinical evidence of a specific disease (Tyas et al., 2007). As many as 41% of octagenarians were found to have at least 3 altered brain proteins (hyperphosphorylated-τ, amyloid-β, α-synuclein, and/or TARD binding-protein 43) (Elobeid et al., 2016). Amyloid-β peptides are one of the proteins most frequently associated with neurodegeneration in both AD and other neurodegenerative conditions including Parkinson's disease (PD) and progressive supranuclear palsy (Noguchi et al., 2005; Goldman et al., 2017; Johar et al., 2017). Even so, the presence of Aβ deposits are also associated with a lower likelihood of healthy aging even in cognitively intact individuals (Knopman et al., 2003; Tyas et al., 2007). Progressive loss of proteostasis and autophagy with aging may prevent the clearance of these disease-promoting toxic aggregates (López-Otín et al., 2013). We can measure Aβ40 and Aβ42 levels in plasma and they have shown high diagnostic and prognostic value in AD (Blennow et al., 2015; Fandos et al., 2017).

Various genetic or lifestyle protective factors have been postulated to explain the mismatch between clinical status (frailty or more specifically cognitive decline) and neuropathological findings. Among them, telomere length has been proposed as a potential prognostic biomarker for disease risk, progression, and premature death in many age-related conditions including neurodegenerative diseases, cancer, stroke and metabolic syndrome (Panossian et al., 2003; Honig et al., 2006; Thomas et al., 2008). Telomere attrition has been observed to accelerate cellular senescence and aging (Donate and Blasco, 2011), but it remains unclear whether it may discern healthy from functionally impaired aging (Saum et al., 2014; Yu et al., 2015; Breitling et al., 2016). This is probably due to difficulties in differentiating functional impairment from frailty, a condition linked to vulnerability to stressors which can be aggravated by concomitant medical and environmental factors (Fried et al., 2001; Mitnitski et al., 2001; Kulminski et al., 2006; Walston et al., 2006).

Among the genetic factors possibly related to cognitive decline in elderls, the presence of the ApoE4 allele is universally considered a risk factor for cognitive decline in AD and other neurodegenerative conditions (Giau et al., 2015). While ApoE4 is long known to be a predictor of longitudinal Aβ accumulation (Lim et al., 2017), it may also play a role in other age-related diseases and longevity (Bonomini et al., 2010; Aboud et al., 2015).

Furthermore, the age-related decline in natural humoral immunity, with a decrease of antibodies able to bind to noxious molecules in atherosclerotic, malignant, or neurodegenerative processes, has been considered as a mechanism to explain the increased risk for chronic diseases in older adults (Rothstein, 2016). Advanced age is characterized by both decreased B-cell numbers and changes in the B-cell repertoire (Bulati et al., 2008). In particular, anti-Aβ antibodies have the capacity to prevent Aβ aggregation-induced neurotoxicity, which is the rationale behind the therapeutic potential of natural anti-Aβ antibodies being explored in clinical trials, though results have been disappointing so far (Szabo et al., 2008; Relkin et al., 2009, 2017; van Dyck, 2018).

Many of these biomarkers have been studied independently in different specific diseases. The general aim of this pilot study was to explore these biomarkers of protein deposition and triggers of cellular senescence and immunological decline in a unique sample of neurologically healthy and functionally impaired (both motor and cognitive) elders, to identify combinations with discriminative value between groups for future in-depth analysis.

## Participants and methods

Presumptively healthy elderly individuals ≥90 years (*n* = 20) were recruited by: (1) searching among family members of hospital workers and patients; and (2) advertising in the local press and social centers. Controls were recruited among institutionalized nonagenarians in nursing homes managed by Matia Fundazioa (*n* = 38). All participants come from a narrow geographical area (Gipuzkoa, Basque Country, Spain).

### Inclusion/exclusion criteria

#### Neurologically healthy nonagenarians

Inclusion criteria for this group were (1) age ≥90 years; and (2) independence in activities of daily living with no significant functional impairment (both cognitive and motor) found in clinical, neurological or neuropsychological assessment.

#### Functionally impaired nonagenarians

Inclusion criteria for this group were (1) age ≥90 years; and (2) dependence and institutionalization because of marked loss in function (cognitive or motor). Clinical diagnoses of underlying clinical conditions were obtained when possible from their electronic medical records at our center and personal records in the institutions. The appropriateness of these diagnoses was validated by an experienced neurologist (ALM) (Supplementary Table 1).

All participants and/or legal representatives gave informed consent using forms approved by Donostia University Hospital's Ethics Committee on the Use of Human Subjects in Research.

### Frailty and dependency assessment

After group allocation, frailty measures were assessed based on medical record data to validate the original clinical classification. Specifically, we used the following three instruments to avoid any controversy in the frailty and dependency classification:

- ***The Canadian Study of Health and Aging (CSHA) Clinical Frailty Scale*** (Rockwood et al., 2005) With this tool, frailty is assessed on a 7-point scale, dividing patients into 4 groups (1–3, very fit-well; 4, vulnerable; 5, mildly frail-dependent for instrumental activities of daily living, and 6–7, moderate-severely frail or dependent for basic activities of daily living), 5 being considered the cut-off for frailty (Rockwood et al., 2005).
- ***The Frailty Index*** (Mitnitski et al., 2001). This approach (described by the same group) considers frailty to result from an accumulation of deficits (20 items) that can be expressed on a continuous scale from 0 to 1, reflecting the proportion of accumulated deficits detected, taken 0.25 as the cut-off for frailty (Rockwood and Mitnitski, 2007). The proportion of deficits for which data were available can be used to calculate a frailty index *(q)* (in our case, data being missing for ≥1 item in most records). For example, if 16 categories could be validated in an individual (the 4 not assessed being considered missing) and 10 of the 16 deficits were present, the frailty index was 10/16 = 0.625.
- ***The Barthel Index*** (Mahoney and Barthel, 1965). This tool measures the extent to which an individual can function independently in her/his basic activities of daily living (feeding, bathing, grooming, dressing, bowel control, bladder control, toilet use, chair transfers, ambulation, and stair climbing). We classified participants using Shah's modified Barthel Index (Shah et al., 1989) as follows: < 20, total dependence; 21–60, severe dependence; 61–90, moderate dependence; 91–99, mild dependence; and 100, full independence.

None of the “neurologically healthy” nonagenarians was classified as frail or dependent, that is, they did not obtain: CSHA Clinical Frailty scale >5, Frailty Index > 0.25, or Barthel Index <61.

#### Clinical, neurological, and neuropsychological assessment

Exclusion of significant comorbidities through a clinical and neurological assessment of healthy nonagenarians was undertaken by an experienced neurologist (ALM) at Donostia University Hospital. Cognitive assessment was performed by two qualified neuropsychologists (MyB and IE) (Supplementary Table 2), to screen for cognitive impairment in the healthy group, ensuring that they were cognitively healthy. Mortality was assessed in October 2017.

### Laboratory assessment

With informed consent, morning blood samples were obtained after overnight fasting, immediately centrifuged, aliquoted, coded, with no reference to group allocation (blinded) and frozen. Specifically, plasma was obtained from EDTA tubes (Vacuntainer, Beckton Dickinson) after centrifugation at 1,250 g for 20 min and plasma was stored at −80°C. Peripheral blood mononuclear cells (PBMCs) were isolated from sodium heparin tubes (Vacuntainer, Beckton Dickinson) following the Ficoll-Hypaque density gradient method, frozen in FSB with 10% DMSO and stored in liquid nitrogen. The following analysis were performed:

- ***Telomere analysis (Experiment 1):*** PBMC telomere length was studied following a validated high-throughput telomere length quantification method (Canela et al., 2007) at the Spanish National Cancer Research Center (CNIO; Maria Blasco's lab).
- ***Quantification of A***β ***peptides in plasma (Experiment 2)*:** plasma Aβ40, Aβ42 and Aβ17 levels were quantified using the corresponding specific enzyme-linked immunosorbent assay (ELISA) kits: ABtest 40, ABtest 42 or ABtest 17 by Araclon Biotech (Zaragoza, Spain). Samples were assayed undiluted and after 1:2 dilution with standard diluent to disrupt Aβ interactions with other plasma components. The free fraction in plasma (FP) of Aβ was quantified by direct analysis of plasma samples, whereas total Aβ in plasma (TP) levels were determined in diluted samples, as previously described (Perez-Grijalba et al., 2015, 2016).
- ***Detection of anti-A***β***40 and A***β***42 antibodies in plasma (Experiment 2):*** Plasma anti-Aβ40 antibody levels were measured at the Araclon Biotech laboratory with ELISAs as previously reported (Lacosta et al., 2018). These assays yielded an estimate of the net adsorbed signal (NAS) defined as the difference between the absorbance observed without pre-adsorption and that observed in the pre-adsorbed sample with the C-terminal peptide of Aβ1-40 or Aβ1-42. To consider that a patient has autoantibodies against Aβ40 or Aβ42 the NAS must be > 3 SD of the technique.
- ***ApoE allele genotype (Experiment 4):*** ApoE alleles were characterized by polymerase chain reaction restriction fragment length polymorphism (PCR-RFLP) analysis of PBMC-derived DNA. The ApoE polymorphic region (227 bp) was amplified with the primers described before by (Wenham et al., 1991). Forward primer: 5′TCC AAG GAG CTG CAG GCG GCG CA3′ and reverse primer: 5′ACA GAA TTC GCC CCG GCC TGG TAC ACT GCC A 3′. The PCR product was then digested with Afl III y Hae II enzymes and restriction fragments separated by gel electrophoresis. The ApoE alleles e3, e2, and e4 were identified by 145 bp, 177 bp and 195 bp bands respectively.

### Data analysis

Using IBM SPSS, both parametric and non-parametric tests were conducted. *P*-values were adjusted for age in a binary logistic regression model. The distribution of participants with and without anti-Aβ40 and Aβ42 antibodies between the groups was assessed with Chi-square tests. Pearson's correlation and receiver operating characteristic (ROC) curve analysis, with the same binary logistic regression model for age adjustment, were used to explore which variables might be good biomarkers of healthy or functionally impaired aging. The ROC curve plots the true positive rate (sensitivity) and false positive rate (1-specificity) as functions of some classifier parameter, allowing to depict the relative trade-offs of each prediction between true positive and false positive. The best possible prediction method would yield a point in the upper left or lower right corner of the ROC space, representing 100% sensitivity (no false negatives) and 100% specificity (no false positives), or a *perfect classification*. A random guess would give a point along a diagonal line (*line of no-discrimination*) from the left bottom to the top right corners.

## Results

Overall, 58 patients were recruited between January 2014 and May 2015: 20 nonagenarians classified as “neurologically healthy” based on clinical, neurological and neurophysiological assessment, and 38 functionally impaired nonagenarians as positive controls. Neurologically healthy nonagenarians were slightly younger and hence, all analysis were adjusted for age. As expected, neurologically healthy nonagenarians systematically scored better in all measures used (CSHA Clinical Frailty Scale, Frailty Index and Barthel Index). Three-year mortality was significantly higher in the functionally impaired nonagenarians (60.52% 23/38 vs. 5% 1/20). Demographic, cognitive. and frailty data are summarized in Table 1.

**Table 1:** Demographic, cognitive, frailty and mortality characteristics of our cohort.

	**Neurologically healthy nonagenarian group (*N* = 20)**	Functionally impaired nonagenarian control group (*N* = 38)^*^	***P***
Age, years	92.37 ± 2.22 SD	94.13 ± 3.66 SD	0.028
Female Sex	13	28	0.49
Frailty Scale (Rockwood): (total n) • Very fit/well • Vulnerable • Dependent For IADL • Dependent For BADL	(20) 15 4 1 0	(35) 0 1 2 32	0.001
Frailty Index (Mitnitski): (total n)	(15)	(36)	
< 0.25	13	0	
Barthel Index			
(total n)	(15)	(14)	
> 61	15	2	0.001
3-year mortality	1	23	0.001
Dementia (total n)	(20)	(33)	
Dementia at inclusion	0	30	0.001

* No valuable information on cognitive status could be obtained from 5 participants in the functionally impaired group.

*Clinical diagnoses are listed in Supplementary Table 1*.

### Experiment 1: Telomere length

**Figure 1:**
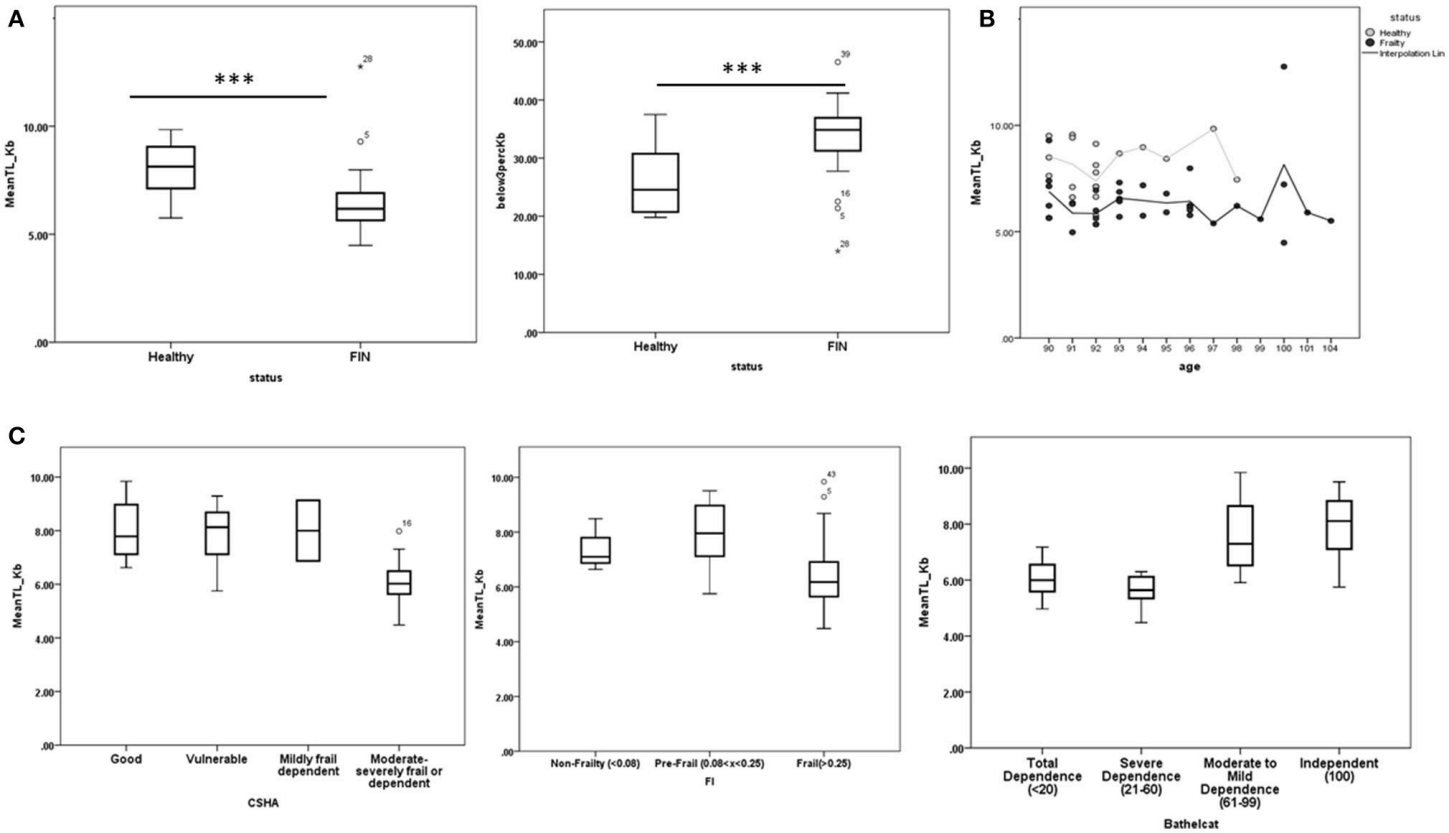
**(A)** Distribution of telomere length and PBMCs with telomere length <3 Kb in the healthy and functionally impaired non-agenarian groups. Healthy nonagenarians showed both higher telomere length and lower percentage of PBMCs with telomere length <3 Kb **(B)** Telomere length in each group distributed by age. No clear relation can be observed. C) Distribution of telomere length after applying each measure of frailty and dependency to all the nonagenarians in the study. Higher frailty or dependency in Canadian Study of Health and Aging scale (CSHA > 5), Frailty index (FI > 0.25), and Barthel Index (Barthelcat < 61) were associated with lower telomere length. ^***^p < 0.001.

Mean PBMC telomere size was significantly higher in the neurologically healthy compared to the functionally impaired group (Figure 1A). In turn, the percentage of PBMCs with short telomere lengths (<3 Kb) was higher in the functionally impaired than in the neurologically healthy group (Figure 1A). The two outliers in Figure 1A (mean TL_Kb vs. Status) correspond to an individual who had leukemia (Size: 12.76kb), which would explain this unusually high value and therefore was excluded from the rest of the analysis and to a patient initially classified as neurologically healthy in the screening phase, but who subsequently developed PD (9.29Kb), falling into the functionally impaired group. No definitive conclusions could be reached when correlating age and telomere length (Figure 1B). We also wanted to check if the above association was exclusive for the functionally impaired status or if it was correlated with other dependence and frailty scales (CSHA, Frailty Index and Barthel Index). Higher frailty or dependency in CSHA (>5), Frailty Index (>0.25), and Barthel Index (< 61) were associated with lower telomere size (Figure 1C).

### Experiment 2: Plasma Aβ40, Aβ42, and Aβ17 peptide levels and immune response to Aβ40 and Aβ42

Levels of all Aβ peptides (circulating free and total in plasma, except for total Aβ17) were lower in healthy than functionally impaired nonagenarians. *P*-values are also shown across all frailty measures (Table 2).

**Table 2:** Aβ levels and ratios in our cohort, and after applying the Clinical Frailty Scale, Frailty Index, and Barthel Index cut-offs (>5, > 0.25, and <61, respectively).

	**Status**					**Clinical frailty scale**					**Barthel index**					**Frailty index**				
**Group**	**Healthy**		**Func. impaired**			**Healthy**		**Func. impaired**			**Healthy**		**Func. impaired**			**Healthy**		**Func. impaired**		
	**mean**	***SD***	**mean**	***SD***	***p***	**mean**	***SD***	**mean**	***SD***	***p***	**mean**	***SD***	**mean**	***SD***	***p***	**mean**	**SD**	**mean**	**SD**	***p***
FP40	131.35	29.39	162.69	42.17	0.004	163.75	13.64	129.65	16.71	0.001	133.19	22.55	141.22	52.34	0.033	155.36	40.43	119.03	22.61	0.132
TP40	299.10	79.58	368.96	95.42	0.007	372.62	98.52	293.26	63	0.001	298.37	69.32	387.46	104.44	0.020	355.49	94.09	243.90	60.14	0.049
FP42	6.29	4.73	10.49	6.10	0.009	10.69	6.32	5.87	3.03	0.001	6.88	5.06	12.65	7.78	0.038	9.45	5.99	6.25	3.13	0.366
TP42	22.53	12.07	31.96	15.05	0.019	32.64	15.48	20.97	9.59	0.001	22.49	13.05	37.72	18.34	0.024	29.90	14.69	15.46	14.07	0.104
FP17	1.32	0.64	2.54	1.98	0.001	2.63	2.04	1.35	0.64	0.001	1.46	1.02	3.03	2.61	0.062	2.28	1.86	1.22	0.81	0.335
TP17	4.47	1.25	5.06	2.31	0.300	5.20	2.37	4.54	1.24	0.277	4.67	1.33	5.27	1.71	0.306	4.99	2.14	4.33	1.20	0.603
FP40/TP40					0.681					0.451					0.479					0.128
FP42/TP42					0.031					0.085					0.320					0.665
FP42/FP40					0.028					0.029					0.094					0.699
TP42/TP42					0.141					0.081					0.086					0.157

Age-adjusted p-values are shown. FP40, FP42 and FP17: free plasma Aβ40, Aβ42 and Aβ17; TP40, TP42 and TP17: total plasma Aβ40, Aβ42 and Aβ17; respectively (pg/ml).

^*^Func. Impaired: Functionally impaired. Red, p < 0.05.

The detection of antibodies against the C-terminal end of Aβ40 and Aβ42 by measuring the NAS with an ELISA kit, showed significant differences between the groups for anti-Aβ40 antibodies, but not for anti-Aβ42 antibodies (including only individuals considered positives for autoantibodies) (Table 3).

**Table 3:** Comparison of net adsorbed signal (NAS) (mean ± SD) of anti-Aβ40 and Aβ42 antibodies between the groups after applying the >3 SD cut-off.

**Group**	**Anti-Aβ*40* NAS**	**Anti-Aβ*42* NAS**
Neurologically healthy	0.211 ± 0.107	0.211 ± 0.068
Functionally impaired	0.147 ± 0.151 (*p* = 0.022)	0.188 ± 0.083 (*p* = 0.396)

In contrast, the distribution of participants with and without anti-Aβ40 antibodies did not differ significantly between the groups (Supplementary Table 5). Furthermore, levels of anti-Aβ antibodies did not correlate with Aβ plasma levels (data not shown).

### Experiment 3: Composite neurologically healthy aging score: searching for a biomarker of healthy aging

We studied the correlations between variables to choose the most informative in the search for a biomarker for aging. Given that mean telomere length and percentage of cells with telomere length < 3 Kb were correlated (Pearson: −0.952), only telomere length was used in subsequent analysis. For amyloid peptides, we considered peptides Aβ42, Aβ40, and Aβ17 as well as the ratio between total (TP42/TP40) and free (FP42/FP40) levels (Supplementary Table 3).

**Figure 2:**
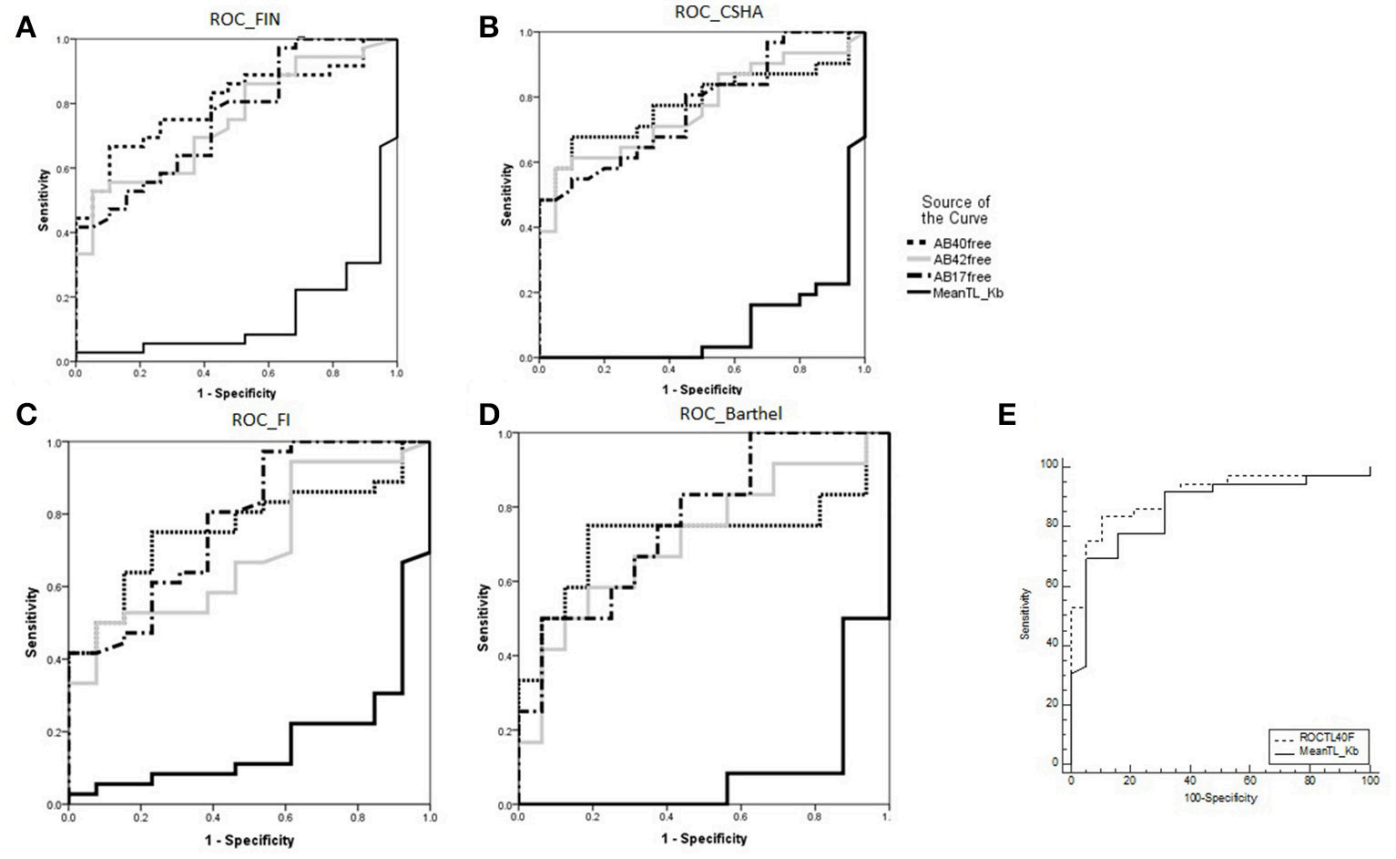
**(A)** ROC curves and areas under the curve (AUCs) for Aβ40, Aβ42, and Aβ17 levels and telomere length for discriminating the functionally impaired non-agenarian group. **(B–D)** The same variables' discriminating power for higher frailty and dependency measured by CSHA, FI and Barthel Index. **E**): “Composite Neurologically Healthy Aging Score”: combined ROC curve and AUC for mean telomere length and total plasma Ab40 (TP40) vs. mean telomere length alone. ^*^AUC for telomere length was 0.133, it being inversely correlated with functionally impaired aging (i.e., functionally impaired nonagenarians are more likely to have shorter telomeres).

Figure 2A shows ROC curves and areas under the curve (AUCs) for telomere length and the Aβ peptides. These were similar after applying all three frailty measures (Figures 2B–D). The ROC curve for the telomere length is particularly striking (AUC = 0.86), showing the highest sensitivity and specificity for discriminating between neurologically healthy and functionally impaired nonagenarians. For a “Composite Neurologically Healthy Aging Score,” we propose using telomere length and TP40, which yielded the highest AUC (0.904) of all combinations assessed (Figure 2E, Supplementary Table 3).

### Experiment 4: ApoE allele genotype

We failed to find a relationship between the apoE allele genotype and healthy status and no ApoE 4/4 allele carriers were found in both groups (Supplementary Table 4).

## Discussion

Telomere shortening is considered one of the primary hallmarks of aging, as it triggers molecular and cellular events underlying aging (López-Otín et al., 2013). It is simultaneously influenced by genetic factors (variants associated with telomerase RNA component loci near the *TERC* gene, and other loci identified from genome-wide studies) (Njajou et al., 2010; Armanios and Blackburn, 2012), and non-genetic factors like the environment (smoking, socioeconomic status, physical activity, fatty acid intake, psychological stressors, and various cardiovascular, endocrine, pulmonary, and skin disorders) (Honig et al., 2006; Cherkas et al., 2008; Thomas et al., 2008; Farzaneh-Far et al., 2010; Nawrot et al., 2010; Gu et al., 2011). In our study, median PBMC telomere length was significantly higher in neurologically healthy nonagenarians, while the percentage of PBMCs with telomere length < 3 Kb was higher in functionally impaired nonagenarians. These results obtained in a small sample of nonagenarians support the hypothesis that telomeres not only decrease with age, but have a prognostic value for detection of frailty and dependence, while longer telomeres are associated with better clinical status in nonagenarians. Telomere shortening could be acting as a trigger of neurodegeneration in the context of previous protein aggregation, as it has been studied in AD or Amyotrophic Lateral Sclerosis (De Felice et al., 2014; Zahn et al., 2015). In further studies, it will be important to determine whether preserved telomeres in healthy individuals are a consequence of protective environmental or genetic factors, or are themselves one of the causes of delayed aging. In relation to this, we did not find a direct relationship between age and telomere length, though this is probably because of the narrow age range of the participants (90–104 years) (Figure 1B).

The presence of lower plasma Aβ peptides levels in neurologically healthy nonagenarians supports the hypothesis that either protein production or clearance in healthy elders differs from functionally impaired controls. There are several reasons to believe this is not entirely related to cognitive decline and AD. First, although functionally impaired controls presented cognitive impairment in a high proportion (78.94% *n* = 30/38), not all of them had a previous diagnose of dementia. Second, in our cohort Aβ peptide levels were more strongly associated with functional impairment than the plasma Aβ42/Aβ40 ratio. Previously, this ratio had been found to be more closely associated with central Aβ peptide levels as well as with cognitive decline in AD subjects (Fandos et al., 2017; Ovod et al., 2017; Nakamura et al., 2018). The fact that we find a stronger association of Aβ peptide levels than their ratios can be most probably influenced by the comparison groups of our study (healthy nonagenarians vs. nonagenarians with functional impairment, not only AD like previously reported). Third, ApoE distribution was not the expected one for the AD population in our community (Alvarez-Alvarez et al., 2003). In previous studies, it has been hypothesized that an abnormality in biomarkers of Aβ deposition is an early event in the disease, before the emergence of either brain atrophy or cognitive symptoms, remaining relatively stable thereafter (Jack et al., 2010). This has also been studied in other neurodegenerative conditions including PD (Goldman et al., 2017; Johar et al., 2017). To clarify this question, it would be interesting to conduct a prospective study with healthy nonagenarians analyzing the rate of onset of cognitive symptoms by monitoring plasma levels of Aβ peptides and anti-Aβ antibodies simultaneously.

Significant differences were observed between both groups when studied for anti-Aβ40 antibody levels suggesting a potential innate protective state which could underlie normal cognition in the oldest old. This would be congruent with the rationale behind several intravenous immunoglobulin (IVIg) clinical trials that initially showed positive effects (Relkin et al., 2009; Kile et al., 2017). Nevertheless, the absence of significant correlation between the presence of anti-Aβ antibodies and plasma amyloid peptide levels suggests that these autoantibodies do not have a marked modulating effect on circulating levels of Aβ, and could possibly be an indirect marker of the age-dependent changes in the B cell compartment previously reported (Bulati et al., 2008). Furthermore, this could explain why other IVIgs failed to meet clinical endpoints in pivotal studies (van Dyck, 2018).

The ApoE allele distribution was similar to that found in a previous study in a healthy Basque population (Alvarez-Alvarez et al., 2003), even though ours is older (90–100 vs. 70–80 years of age in the aforementioned study). Interestingly, this distribution did not differ between functionally impaired and neurologically healthy nonagenarians, and as we mentioned earlier, functionally impaired nonagenarians in our study did not have a similar ApoE allele distribution to that in AD patients in the Basque Country. These supports the fact that the functionally impaired group in this study was not only “cognitively impaired,” but frail and dependent because of cognitive and motor impairment. As indicated in Supplementary Table 4, no ApoE 4/4 allele carriers were found. ApoE4 promotes the accumulation of Aβ (Zhao et al., 2014), but it remains unclear whether it confers a risk of developing dementia or only determines the age of onset, as previously suggested (Khachaturian et al., 2004). Our results are congruent with previous studies showing that the effect size of the ApoE gene on dementia was dramatically lower in over-75-year-olds (Lacour et al., 2017).

In the search for a combined biomarker of neurologically healthy aging in the oldest old, we found that the combination of mean telomere length and TP40 had the strongest discriminative power between both groups of nonagenarians, as it yielded the highest AUC of all markers measured independently or in combination (Supplementary Table 3). We call this combined variable the “Composite Neurologically Healthy Aging Score” and its ROC curve is shown in Figure 2E. In synthesis, these results support the hypothesis that protein aggregation in brains may not be sufficient for neurodegeneration, and that increased protective factors against accelerated aging in combination with decreased markers of pathological changes in brain tissue may prevent neurodegeneration.

It should be noted that there are several limitations in this preliminary approach that could influence our results. First of all, the definition of “neurologically healthy aging” is subjective. Although low frailty status may reflect healthy aging, there are numerous frailty indices and scales, and each of them takes different variables into account (e.g., dependence, cognitive status, mobility, and comorbidity), underlining the difficultly of reaching an agreed definition of this concept. This variability in defining poor aging, frailty and functional dependence is probably contributing to the controversy around the potential of telomere length preservation to predict healthy aging. In relation to this, we have compared cognitive and functionally healthy nonagenarians with nonagenarians whose comorbidities and poor or frail aging have led them to a decline in functioning, frailty and dependence, and this is the cornerstone of our study. To avoid bias from this “subjectivity,” blood sample studies were blinded to the participants' categorization. Secondly, our sample size is small, due to the small number of the population that reaches such old ages, and though statistically significant values are valid, the number of individuals enrolled in this study could have hindered our ability to find associations with plasma anti-Aβ antibodies. Lastly, it is worth mentioning that due to the biochemical nature of Aβ peptides (and particularly, their extreme hydrophobicity), these can be found free in plasma, and bound to plasma components (total level). Thus, any further Aβ blood tests should consider the amount of peptides in both of these fractions, as measured in the present work.

## Conclusion

In conclusion, in the studied cohort, neurologically healthy non-agenarian individuals had longer telomere lengths and lower levels of Aβ40 (total and free), Aβ42 (total and free), and free Aβ17 in plasma than functionally impaired nonagenarians. We introduce a score, the “Composite Neurologically Healthy Aging Score,” which shows better discrimination between neurologically healthy and functionally impaired nonagenarians than each of the independent markers alone. The elevated level of natural anti-Aβ40 antibodies present in some healthy nonagenarians may suggest a protective effect against cognitive decline and poor aging. These associations should be confirmed in larger cohorts before application in future clinical therapeutic or preventive trials. Moreover, it is necessary to confirm the predictive value of the score in a wider elder population in order to stratify the risk of poor aging for interventional actions in the oldest old.

## Ethics statement

This study was carried out in accordance with the recommendations of CEIC of Euskadi (Basque Country) with written informed consent from all subjects. All subjects gave written informed consent in accordance with the Declaration of Helsinki. The protocol was approved by the CEIC of Euskadi.

## Author contributions

GF-E, MiZ, JY, BI, FM, IV and ALM contributed to the recruitment and clinical and neurological characterization of subjects. MyB and IE contributed to the neuropsychological characterization. MG, NS, and MB contributed to the telomere studies. AL, JC, VP-G, NF, PP, and MS contributed to the amyloid and anti-amyloid antibodies study. AA, MM-C, and DO contributed to the ApoE study. GF-E, AA, MoZ, DO, and ALM contributed to the writing and reviewing of this manuscript.

### Conflict of interest statement

The authors declare that the research was conducted in the absence of any commercial or financial relationships that could be construed as a potential conflict of interest.
