# Supplementary material for: Blood Markers in Healthy-Aged Nonagenarians: A Combination of High Telomere Length and Low Amyloidβ Are Strongly Associated With Healthy Aging in the Oldest Old
Source: Front Aging Neurosci. 2018 Nov 28;10:380. doi: 10.3389/fnagi.2018.00380 (PMC6280560; doi:10.3389/fnagi.2018.00380)
Supplement: Supplementary file 1 [file Table_1.pdf]

Supplementary Table 1

13-616	Cognitive impairment<br>Myasthenia gravis	Cardiac dysfunction	Vascular risk factors	Chronic renal insufficiency	Musculoskeletal pathology	Sensory impairment	Anaemia
14-723	Cognitive impairment			Chronic renal insufficiency	Musculoskeletal pathology	Sensory impairment	
14-724	Cognitive impairment	Cardiac dysfunction	Vascular Risk Factors	Chronic renal insufficiency	Musculoskeletal pathology	Sensory impairment	
14-725	Cognitive impairment	Cardiac dysfunction	Vascular Risk Factors		Musculoskeletal pathology	Sensory impairment	
14-726	Polyneuropathy				Musculoskeletal pathology	Sensory impairment	Anaemia
14-727	Cognitive impairment	Cardiac dysfunction	Vascular Risk Factors				
14-729	Cognitive impairment		Vascular Risk Factors				
14-730	Cognitive impairment	Cardiac dysfunction			Musculoskeletal pathology	Sensory impairment	
14-731	Cognitive impairment<br>Stroke	Cardiac dysfunction		Chronic renal insufficiency			
14-732	Cognitive impairment<br>Stroke	Cardiac dysfunction	Vascular Risk Factors		Musculoskeletal pathology	Sensory impairment	Anaemia<br>COPD
14-733	Cognitive impairment		Vascular Risk Factors	Chronic renal insufficiency	Musculoskeletal pathology		

14-734	Cognitive impairment			Chronic renal insufficiency		Sensory impairment	
14-736	Cognitive impairment	Cardiac dysfunction	Vascular Risk Factors			Sensory impairment	Cancer
14-737	Cognitive impairment				Musculoskeletal pathology	Sensory impairment	Anaemia
14-738	Cognitive impairment	Cardiac dysfunction		Chronic renal insufficiency			Anaemia
14-739	Cognitive impairment	Cardiac dysfunction	Vascular Risk Factors	Chronic renal insufficiency			Anaemia COPD
14-740	Cognitive impairment				Musculoskeletal pathology	Sensory impairment	
14-741	Cognitive impairment	Cardiac dysfunction	Vascular Risk Factors	Chronic renal insufficiency		Sensory impairment	
14-780	Cognitive impairment	Cardiac dysfunction					Cancer
14-781	Cognitive impairment	Cardiac dysfunction		Chronic renal insufficiency			Hypothyroidism
14-782	Cognitive impairment Stroke	Cardiac dysfunction	Vascular Risk Factors		Musculoskeletal pathology	Sensory impairment	
14-783	Cognitive impairment		Vascular Risk Factors			Sensory impairment	
14-784	Cognitive impairment		Vascular Risk Factors		Musculoskeletal pathology		

14-785	Cognitive impairment						
14-786		Cardiac dysfunction	Vascular Risk Factors	Chronic renal insufficiency		Sensory impairment	
14-791	Cognitive impairment Epilepsy	Cardiac dysfunction					
14-792	Hydrocephalus Myelopathy		Vascular Risk Factors			Sensory impairment	COPD
14-793	Cognitive impairment				Musculoskeletal pathology	Sensory impairment	
14-794	Stroke					Sensory impairment	
14-795	Cognitive impairment	Cardiac dysfunction	Vascular Risk Factors			Sensory impairment	Hypothyroidis m
14-820*	Parkinson’s Disease						Cancer
14-821	Cognitive impairment	Cardiac dysfunction		Chronic renal insufficiency			Anaemia
14-822		Cardiac dysfunction		Chronic renal insufficiency		Sensory impairment	
14-823		Cardiac dysfunction	Vascular Risk Factors				Cancer
14-824	Cognitive impairment Myelopathy			Chronic renal insufficiency			COPD
09-1045	Parkinson’s Disease					Sensory impairment	
10-953	Cognitive impairment		Vascular	Chronic renal		Sensory	Anaemia

			Risk Factors	insufficiency		impairment	
14-175	Cognitive impairment Parkinson's Disease	Cardiac dysfunction					COPD Cancer

Supplementary Table 1: Functionally impaired nonagenarians comorbidity:

- Cardiac dysfunction: arrhythmia, cardiac insufficiency, ischemia, valvulopathy
- Vascular risk factors: high blood pressure, diabetes mellitus, dyslipidaemia
- Musculoskeletal pathology: arthrosis, osteoporosis, rheumatic polymyalgia
- Sensory impairment: vision or hearing loss

*Patient suffering from leukaemia, with abnormally high telomere values

+Healthy nonagenarians did not present any relevant comorbidity (table not shown)
