# Supplementary material for: Blood Markers in Healthy-Aged Nonagenarians: A Combination of High Telomere Length and Low Amyloidβ Are Strongly Associated With Healthy Aging in the Oldest Old
Source: Front Aging Neurosci. 2018 Nov 28;10:380. doi: 10.3389/fnagi.2018.00380 (PMC6280560; doi:10.3389/fnagi.2018.00380)
Supplement: Supplementary file 2 [file Table_2.pdf]

Supplementary Table 2

Mini-Mental State Examination (MMSE, Folstein et al., 1975)
Boston Naming Test (BNT, Goodglass and Kaplan, 2000)
Phonemic and Semantic Verbal Fluency (Peña Casanova et al., 2009)
Free and Cued Selective Reminding Test (FCSRT, Buschke, 1984).
Digit Span (WMS-III, Weschler, 1997)
CERAD Word List Memory (Rosen, Mohs, & Davis, 1984)
Clock Drawing Test (Mendez et al. 1992)
Trail Making Test (Parts A & B, Reitan, 1955)

Supplementary Table 2: Neuropsychological assessment: cognitive test.
