# Supplementary material for: Blood Markers in Healthy-Aged Nonagenarians: A Combination of High Telomere Length and Low Amyloidβ Are Strongly Associated With Healthy Aging in the Oldest Old
Source: Front Aging Neurosci. 2018 Nov 28;10:380. doi: 10.3389/fnagi.2018.00380 (PMC6280560; doi:10.3389/fnagi.2018.00380)
Supplement: Supplementary file 3 [file Table_3.pdf]

Supplementary Table 3:

	Mean TL	Free AB40	Total Ab40	Free Ab42	Total Ab42	Free Ab17	Total Ab17	Acp40	Acp42	$Ab42_{free}/42_{tot}$ ratio	$Ab42_{free}/40_{free}$ ratio
**Mean TL**	**0.86**										
**Free AB40**	0.883	**0.801**									
**Total Ab40**	0.904	0.759	**0.786**								
**Free Ab42**	0.879	0.761	0.726	**0.748**							
**Total Ab42**	0.892	0.761	0.732	0.714	**0.736**						
**Free Ab17**	0.892	0.755	0.726	0.736	0.739	**0.76**					
**Total Ab17**	0.874	0.761	0.753	0.732	0.689	0.77	**0.597**				
**Acp40**	0.881	0.922	0.817	0.824	0.784	0.791	0.712	**0.625**			
**Acp42**	0.911	0.672	0.813	0.656	0.688	0.688	0.641	1	**0.5**		
**$Ab42_{free}/42_{tot}$ ratio**	0.87	0.856	0.784	0.758	0.752	0.784	0.752	0.824	0.571	**0.778**	
**$Ab42_{free}/40_{free}$ ratio**	0.873	0.763	0.726	0.761	0.679	0.738	0.661	0.771	0.656	0.625	**0.66**

Supplementary Table 3: Table showing the different possible combined areas under the curve (AUCs), Acp40 and 42 values have been discarded because of small sample size (6 patients). Red font indicates the combined AUCs better than the individual AUC value. The black square indicates the best AUC obtained.
