# Supplementary material for: Blood Markers in Healthy-Aged Nonagenarians: A Combination of High Telomere Length and Low Amyloidβ Are Strongly Associated With Healthy Aging in the Oldest Old
Source: Front Aging Neurosci. 2018 Nov 28;10:380. doi: 10.3389/fnagi.2018.00380 (PMC6280560; doi:10.3389/fnagi.2018.00380)
Supplement: Supplementary file 4 [file Table_4.pdf]

Supplementary Table 4:

			ApoE			Total
			3/3	2/4	3/4	
Status	Healthy	Count	15	2	3	20
		% within status	75.0%	10.0%	15.0%	100.0%
	Frailty	Count	30	3	4	37
		% within status	81.1%	8.1%	10.8%	100.0%
Total		Count	45	5	7	57
		% within status	78.9%	8.8%	12.3%	100.0%

Supplementary Table 4: No relationship was found between ApoE allele genotype and healthy ageing status.
