# Supplementary material for: Blood Markers in Healthy-Aged Nonagenarians: A Combination of High Telomere Length and Low Amyloidβ Are Strongly Associated With Healthy Aging in the Oldest Old
Source: Front Aging Neurosci. 2018 Nov 28;10:380. doi: 10.3389/fnagi.2018.00380 (PMC6280560; doi:10.3389/fnagi.2018.00380)
Supplement: Supplementary file 5 [file Table_5.pdf]

Supplementary Table 5:

Distribution of participants with/without anti-Aβ40 antibodies

	**Anti-Aβ40 antibody negative participants**	**Anti-Aβ40 antibody positive participants**	Total	**% Anti-Aβ40 antibody positive**
Functionally impaired	28	10	38	26.3
Healthy	14	6	20	30
Total	42	16	58	27.6

p=0.765

Distribution of participants with/without anti-Aβ42 antibodies

	**Anti-Aβ42 antibody negative participants**	**Anti-Aβ42 antibody positive participants**	Total	**% Anti-Aβ42 antibody positive**
Functionally impaired	21	17	38	44.7
Healthy	11	9	20	45
Total	32	26	58	44.8

p=0.985

Supplementary Table 5: Chi-Squ are test showed no significant differences in the distribution of individuals with or without anti-Aβ40 (p=0.765) or Aβ42 (p=0.985) antibodies between functionally impaired and healthy groups.
